# Synthesis and Characterization of Mesoporous Aluminum Silicate and Its Adsorption for Pb (II) Ions and Methylene Blue in Aqueous Solution

**DOI:** 10.3390/ma15103562

**Published:** 2022-05-16

**Authors:** Hye-Soo Jo, Hyeonjin Kim, Seog-Young Yoon

**Affiliations:** Department of Materials Science and Engineering, Pusan National University, Busan 46241, Korea; hyesoo@pusan.ac.kr (H.-S.J.); h.kim@pusan.ac.kr (H.K.)

**Keywords:** aluminum silicate, mesoporous, low density powder, adsorption

## Abstract

Aluminum silicate powder was prepared using two different syntheses: (1) co-precipitation and (2) two-step sol-gel method. All synthesized powders were characterized by various techniques including XRD, FE-SEM, FT-IR, BET, porosimeter, and zetasizer. The particle morphology of the synthesized aluminum silicate powder was greatly different depending on the synthesis. The synthesized aluminum silicate powder by co-precipitation had a low specific surface area (158 m^2^/g) and the particle appeared to have a sharp edge, as though in a glassy state. On the other hand, synthesized aluminum silicate powder by the two-step sol-gel method had a mesoporous structure and a large specific surface area (430 m^2^/g). The aluminum silicate powders as adsorbents were characterized for their adsorption behavior towards Pb (II) ions and methylene blue in an aqueous solution performed in a batch adsorption experiment. The maximum adsorption capacities of Pb (II) ions and methylene blue onto the two-step sol-gel method powder were over four-times and seven-times higher than that of the co-precipitation powder, respectively. These results show that the aluminum silicate powder synthesized with a two-step sol-gel method using ammonia can be a potential adsorbent for removing heavy metal ions and organic dyes from an aqueous solution.

## 1. Introduction

Water pollution caused by pollutants including heavy metals, organic dyes, pigments, and microplastics which are discharged from various industrial activities has become a serious global problem and aroused tremendous attention [1,2,3]. Meanwhile, heavy metals are discharged from various industries such as electroplating, batteries, welding, and printed circuit boards to flow underwater [4,5]. Heavy metals in wastewater are one of the most widely known harmful pollutants [6]. Some heavy metal ions, such as lead, cadmium, and mercury, are highly toxic even at low ion concentrations and are not biodegradable, which is very harmful when returned to humans by ecosystem circulation [3,4]. Therefore, it is urgent to remove the harmful substances from wastewater to minimize the negative effect on the environment [6]. Several methods have been used to resolve this problem, including membrane filtration, photodegradation, ion exchange, adsorption, and chemical precipitation [7,8]. Among the various methods for wastewater purification, adsorption has been widely used for its advantages such as efficiency and safety as well as economic [9]. In addition, there is an advantage that various types of adsorbents having various functional groups can be synthesized [10].

In general, activated carbons and aluminosilicate-based zeolites are some of the widely used adsorbents for removing pollutants from aqueous solutions [11]. However, zeolite is effective in removing heavy metal ions but has disadvantages in removing organic dyes due to its long adsorption time and low adsorption capacity, and activated carbon has the advantage of high adsorption capacity but is limited in use due to its high cost and need for regular regeneration [1,12]. Recently, mesoporous silica materials with a large specific surface area and high pore volume have been widely used to remove pollutants from wastewater [13]. However, as the consumption of silica materials increased on a large scale in the industry, the cost of silica materials using commercial silica precursors increased relatively highly [14]. Therefore, it is highly desirable to realize the production of a new type of material to replacing mesoporous silica [11].

Aluminum silicate is considered as one of the ideal materials for would be a candidate as an adsorbent that can be obtained from inexpensive precursors [6]. The structural characteristic aluminum silicate consists of one Si atom and four O atoms forming a tetrahedron, and Al atoms forming a network surrounded by O atoms [6]. Al atoms could be decomposed to exist in an ionic state, and O bonded to Al binds to surround hydrogen ions to generate a hydroxyl group (-OH) [15]. Therefore, there are many silanol groups (Si-OH) related to physical adsorption on the aluminum silicate surface [16]. It is very hard to find articles related to synthetic aluminum silicate adsorbents even though several studies have been reported on natural or modified aluminum silicate adsorbents [1,7,11,17].

In this study, aluminum silicate powders were synthesized using two different syntheses methods such as co-precipitation and sol-gel method. In the sol-gel method, the process was divided into two steps using ammonia. The two-step sol-gel method was selected to form particles with a uniform size and the advantage of being synthesized at a low temperature. The synthesized powders obtained with two different methods were compared and characterized in terms of crystallinity, particle morphology, particle size, and specific surface area. The adsorption performance of the synthesized aluminum silicate powder was characterized by the adsorption capacity and removal efficiency of Pb (II) ions in heavy metal ions and methylene blue in organic dyes.

## 2. Materials and Methods

### 2.1. Synthesis of Aluminum Silicate Powder

Sodium silicate solution (SSS, Na_2_SiO_3_) was purchased from DAEJUNG with a pH value of 11.0–11.5. The solution contained 9–10% Na_2_O and 28–30% SiO_2_. Aluminum nitrate nonahydrate (Al(NO_3_)_3_ 9H_2_O, SAMCHUN, >98.0%) was used as an aluminum precursor and had a pH value of 2–4. Ammonia solution (NH_4_OH, 28–30%) extra pure was purchased from JUNSEI. The metering pump used for precipitation was LEAD FLUID’s BT101L flow tube pump, YT15, and silicone tubing.

**Figure 1:**
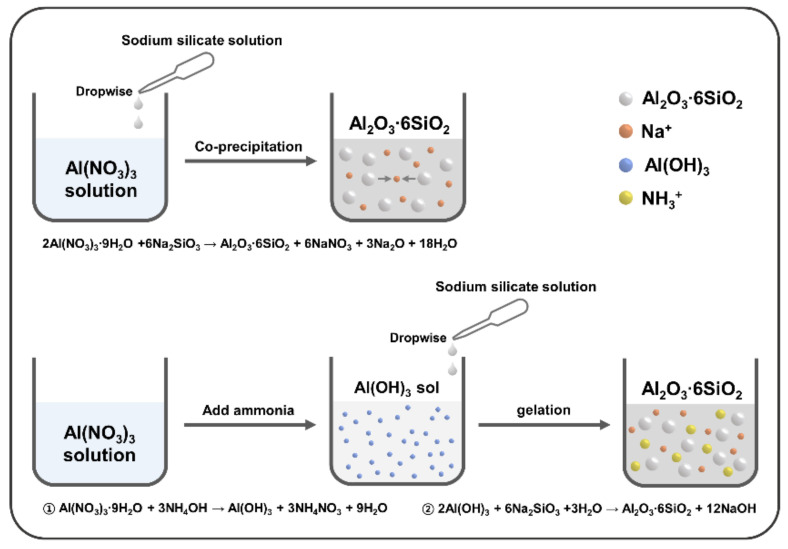
Schematic illustration of experimental procedure for synthesizing the aluminum silicate powder.

For the synthesis of aluminum silicate powder, precursor solutions were prepared: 10 mL SSS was diluted with 90 mL of distilled water and 6.034 g of aluminum nitrate nonahydrate with deionized (DI) water was stirred until a clear solution was obtained. Figure 1 shows the overall experimental procedure for synthesizing the aluminum silicate powder. As shown in Figure 1, in the case of co-precipitation, the aluminum silicate powder was prepared by directly dropping (7.5 mL/min) of sodium silicate solution onto the aluminum nitrate solution. Meanwhile, aluminum silicate powders were synthesized in the two-step sol-gel method. In the first step, an ammonia solution with different ammonia contents (2%, 4%, 6%, 8%, 10%, and 12%) was added to the aluminum nitrate solution to prepare an aluminum hydroxide sol. In the second step, a gel was generated by dropping the sodium silicate solution into a prepared sol for synthesizing mesoporous aluminum silicate powder. The final solution was stirred for 30 min, after the reaction was completed, it was washed with 1300 mL of distilled water using a vacuum filtration device to remove unreacted substances. After filtration, dried at 80 °C for 24 h and pulverized to fine powders. The samples with different ammonia contents were notated as shown in Table 1.

### 2.2. Characterization

X-ray diffraction patterns (XRD, SmartLab SE, Rigaku Corp., Tokyo, Japan) were obtained to identify the phase of the synthesized aluminum silicate powder using CuKa radiation (1.540562 A). The diffractometer operated at 40 kV, 40 mA, and a step size of 0.01 was used. Infrared spectra of the synthesized aluminum silicate powder were obtained using a Fourier transform infrared spectroscopy (FTIR, Nicolet iS50 Spectrometer, Thermo Fisher Scientific, Waltham, MA, USA). The powders were mixed with KBr and pressed into a pellet. The absorption spectra were recorded in the region 400 to 4000 cm^−1^, by collecting 16 scans at 4 cm^−1^ resolution. The spectral analysis was performed by using the Spectragraph software. The particle size of the synthesized aluminum silicate powder was measured through a particle size analyzer (PSA, LS 13 320, Beckman Coulter, Brea, CA, USA). It measures the size distribution of suspension particles in a liquid or dry powder form using dynamic light scattering. The morphology of the synthesized aluminum silicate powder was observed by Field-Emission Scanning Electron Microscopy (FE-SEM, MIRA 3, TESCAN, Brno, Czech Republic). Measurements of the specific surface area for synthesized aluminum silicate powder were taken using a surface area analyzer (ASAP 2420, Micromeritics, Norcross, GA, USA). The method employed was the adsorption of high purity nitrogen. The measurements involved determining the isotherms of nitrogen adsorption at liquid nitrogen temperature (77.3 K) and calculating the monolayer capacity based on the BET (Brunauer, Emmett, and Teller) adsorption isotherm. The porosity of the synthesized aluminum silicate powder was obtained using a porosimeter (AutoPore IV 9500, Micromeritics, Norcross, GA, USA). The zeta potential of the synthesized aluminum silicate powder was measured using Zetasizer (Zetasizer Nano ZSP, Malvern Panalytical, Malvern, UK), and electrophoretic light scattering was used for the measurement.

### 2.3. Pb (II) Ion Adsorption Test

Lead (II) nitrate (Kanto Chemical Co., Inc., Tokyo, Japan) was used for adsorption of heavy metal ions. To investigate the Pb (II) ion adsorption performance of the synthesized aluminum silicate, 2 g of Pb(NO_3_)_2_ and 1 L of distilled water were stirred to prepare a solution. The pH adjustment was determined to be 8 through a prior experiment and was adjusted using ammonia. An amount of 0.1 g each of aluminum silicate powder was added to 25 mL Pb (II) solution, and after 90 min of reaction, only 10 mL of the supernatant was separated by centrifugation for 30 min and analyzed using an inductively coupled plasma atomic emission spectrophotometer (ICP-AES, ACTIVA-S, JY HORIBA, Kyoto, Japan).

### 2.4. Methylene Blue Adsorption Test

Methylene blue solution (0.1%) of DAEJUNG was used for dye adsorption. To observe the methylene blue adsorption performance of the synthesized aluminum silicate powder, a methylene blue solution of 100 ppm was prepared using a 0.1% methylene blue solution. In 100 ppm methylene blue 40 mL solution, 0.02 g each of aluminum silicate powder was added and reacted for 50 min. After that, only 10 mL of the supernatant was separated by centrifugation and analyzed using Ultraviolet-Visible Spectroscopy (UV-Vis, V-730, JASCO Inc., Easton, MD, USA) at 664 nm.

## 3. Results and Discussion

### 3.1. Characterization of the Synthesized Powder

**Figure 2:**
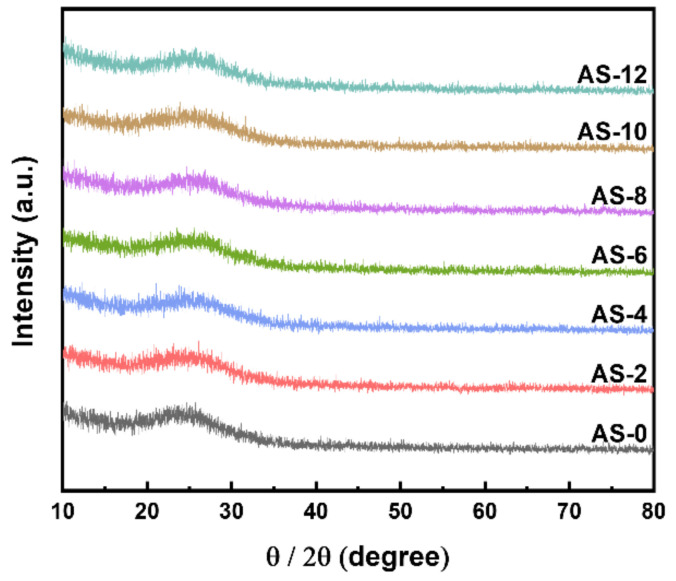
X-ray diffraction patterns of the synthesized aluminum silicate powder with different ammonia contents.

XRD patterns for the synthesized powders with different ammonia contents are shown in Figure 2. As can be seen in Figure 2, all synthesized powder had a broad peak around 25 degrees, which is indicative of the amorphous phase. These results are in good agreement with the previously reported results of the amorphous kaolin in the study of synthesized aluminum silicate [6,18,19]. Moreover, the phase and secondary phase of the synthesized aluminum silicate with amorphous were not affected by the ammonia contents.

**Figure 3:**
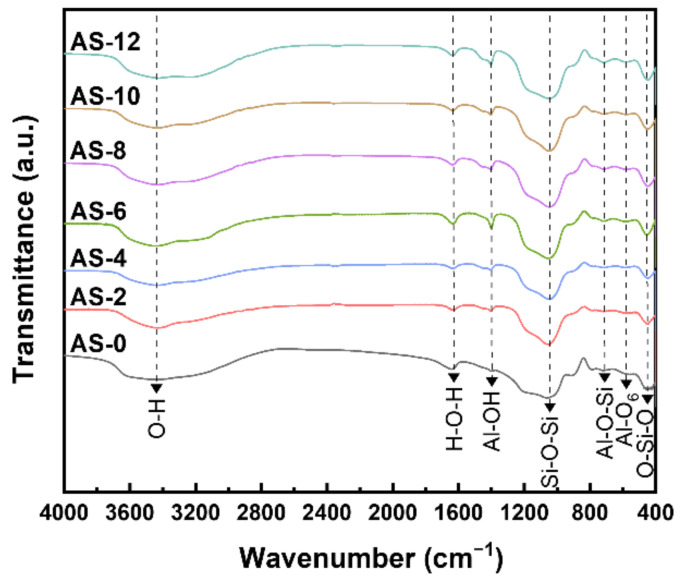
IR spectra of the synthesized aluminum silicate powder with different ammonia contents.

The FT-IR spectroscopy was used to further confirm that the aluminum silicate powder was properly synthesized whether adding ammonia or not. Figure 3 and Table 2 show the FT-IR spectra and values of the synthesized powder with different ammonia contents. All powders display almost similar characteristic bands of aluminum silicate framework, including absorbance band at around 700 cm^−1^ corresponding to the stretching vibration of Al-O-Si bond [19,20]. To explain the reason why Al-O-Si bond was formed, when a sodium silicate solution is dropped dropwise in a state where an aluminum hydroxide sol is generated using ammonia as a catalyst, the siloxane bond of sodium silicate is dissociated by hydroxide ions of the sol [21,22]. This is because the dissociated siloxane bonds react with aluminum ions to form an Al-O-Si bond, and as sodium silicate is added, more aluminum ions are incorporated into the silicate network in the sol [21,23]. The absorption band located at 455 cm^−1^ shows the rocking of the O-Si-O bond, and the absorption band of the Al-O_6_ bond at 580 cm^−1^ is assigned to the bending and stretching vibrations [19,20,24]. The Si-O-Si bond shows stretching vibration mode at 1045 cm^−1^, and the Al-OH bond shows bending vibration at 1400 cm^−1^ [19,20,25]. The absorption band located at 1630 cm^−1^ represents stretching vibration with physically adsorbed water molecules, and the broad absorption band at 3440 cm^−1^ represents O-H with bending and stretching vibrations [11,19,20]. These findings combined with the results of XRD diffraction patterns for the synthesized powders demonstrate that all of the synthesized powder properly formed to be aluminum silicate network. In general, the particle size of aluminum silicate powder would lie in 10–30 μm for well flowability as the absorbent. Table 3 represents the particle size of the synthesized aluminum silicate powder with different ammonia contents. As shown in Table 3, the mean particle sizes of all powder were ranged between 10 and 30 μm, which is a suitable particle size as the adsorbent [26,27]. Particularly, the mean particle size of the AS-0 was a low value of around 12.76 μm compared to the aluminum silicate powder synthesized with two-step sol-gel method. Likewise, the particle size of the synthesized aluminum silicate powder gradually increased as the content of ammonia increased. This phenomenon could be explained that pH increases due to the increase in ammonia content, the decomposition of the silanol groups on the surface of aluminum silicate is accelerated, and water adsorbed on the surface is released to form siloxane bonds [28]. As a result, particles agglomerate and the particle size increase as the ammonia content increases.

**Figure 4:**
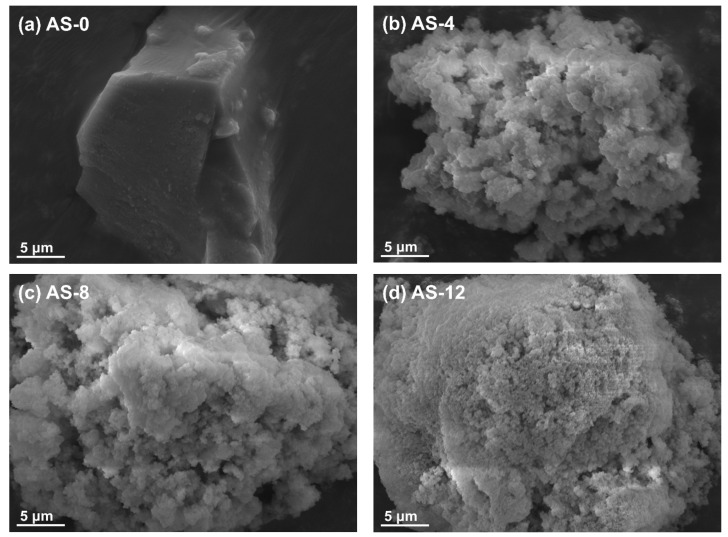
SEM images of the synthesized aluminum silicate powder according to different ammonia contents: (**a**) AS-0, (**b**) AS-4, (**c**) AS-8, and (**d**) AS-12.

**Figure 5:**
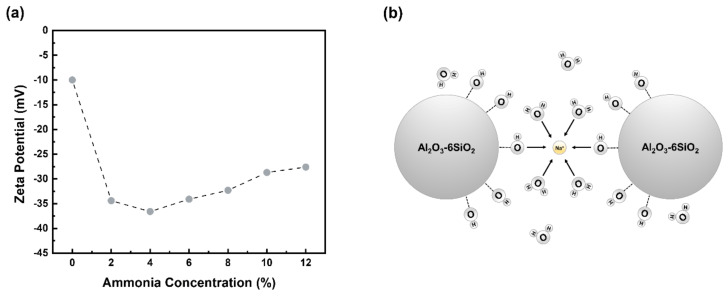

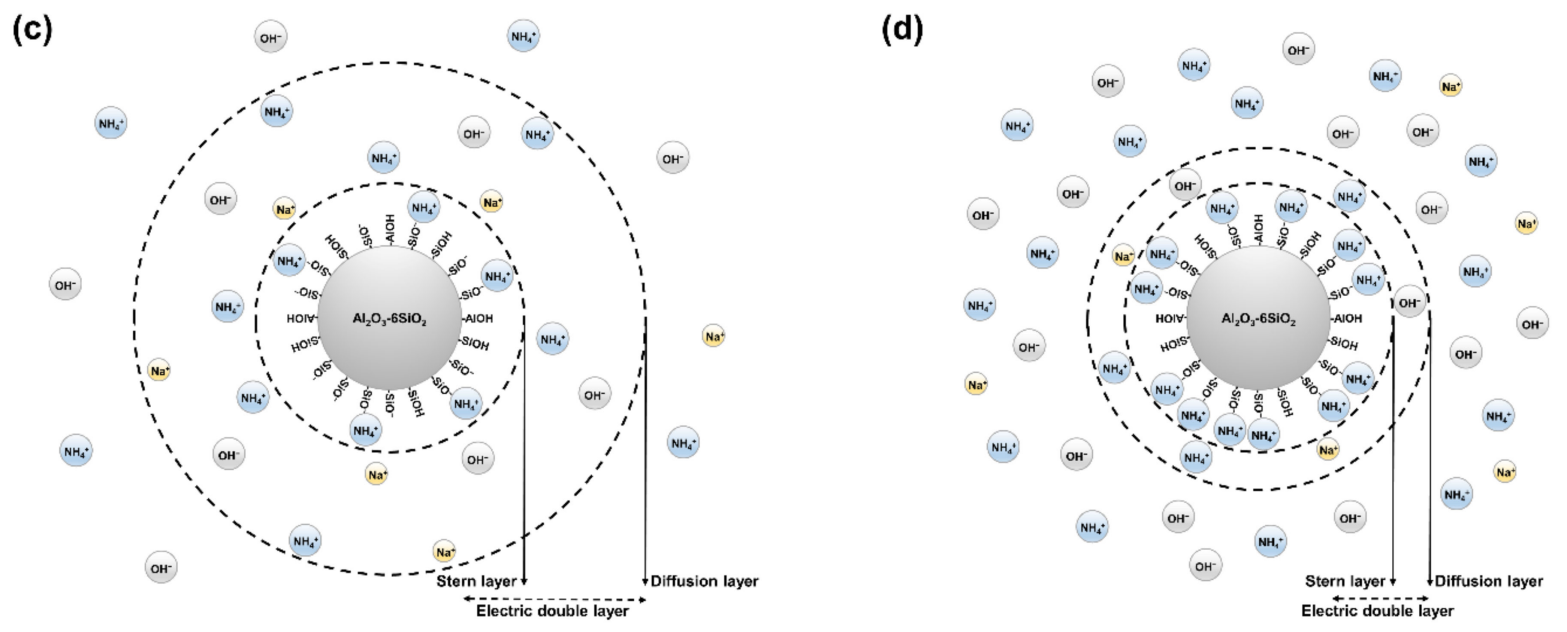
(**a**) Zeta potential value of aluminum silicate in a solution with different ammonia contents, schematic picture of (**b**) aggregation behavior with the particle of AS-0 powder, change in the thickness of the electrical double layer when (**c**) the content of ammonium ions is low, and (**d**) the content of ammonium ions is high.

The morphology and mesostructure of the synthesized aluminum silicate with different ammonia contents were evaluated by using the FE-SEM. The FE-SEM images of the synthesized aluminum silicates are illustrated in Figure 4. As shown in Figure 4a, the particle morphology of the synthesized aluminum silicate by co-precipitation was highly condensed and the surface of the particle appeared to have a sharp edge, as though in a glassy state. In contrast, when synthesized by the two-step sol-gel method, the aluminum silicate powders appeared to be aggregated and agglomerated with lots of small particles, and had a mesoporous surface as shown in Figure 4b–d. In addition, as the content of ammonia increased the mesoporous surface of aluminum silicate particles changed to become a denser structure. These phenomena can be explained by measuring the zeta potential, which is a parameter used as an index to evaluate dispersion stability by indicating the amount of surface charge of particles suspended in a solvent as a value [29,30]. Therefore, the higher the absolute value of the zeta potential, the more stable the particles [31]. Figure 5a illustrates the results of measuring the zeta potential values of the synthesized aluminum silicate with different ammonia contents. As can be seen in Figure 5a, the zeta potential was steeply increased with adding the ammonia, and then its value was not much changed with the ammonia contents.

In the case of AS-0 (by co-precipitation), the zeta potential is very low, indicating that the particles would be very unstable state. Their behavior under solution can be explained as shown in Figure 5b. The Na^+^ ions in the colloidal aluminum silicate act as bridging ions because of their small size. First, when Na^+^ ion is adsorbed on the surface of aluminum silicate particle, the Na^+^ ion is surrounded by oxygen atoms of six adjacent water molecules. At this time, some of the oxygen atoms of water molecules may be replaced by oxygen atoms of the silanol group (Si-OH) present on the particle surface. The oxygen atom of the silanol group is directly connected to the Na^+^ ion. When this Na^+^ ion collides with another aluminum silicate particle, it coordinates with the oxygen atom of the silanol group [32]. Then the two aluminum silicate particles strongly aggregate, forming the siloxane (Si-O-Si) groups and water [28,33,34]. With the addition of ammonia, the zeta potential was not much changed, but its value had a maximum value at 4% (AS-4) and then gradually decreased with the increase in ammonia. These variations would affect the formation of particle size and morphology during the reaction process for synthesizing the aluminum silicate powder in the aqueous solution. The value of the zeta potential is proportional to the thickness of the electric double layer, where the thickness of the electric double layer decreases as the electrolyte ions increases, as represented in equation (1) [35,36].

(1) $$\kappa^{-1}=\sqrt{\frac{\varepsilon \varepsilon_{0}k_{B}T}{2ze^{2}\rho_{0}}\;\;\;}$$

Here, *κ*^−1^ is the thickness of the electric double layer, *ρ*_0_ represents the concentration of ions, *e* represents the elementary charge, *ε* represents the relative permittivity of the solution, *ε*_0_ represents the vacuum permittivity, *z* represents the counter ion’s valence, *k_B_* represents the Boltzmann’s constant, and *T* represents the absolute temperature.

As shown in Figure 5c, when the content of ammonium ions is low, the ammonium ions do not sufficiently shield the negative charge on the surface of aluminum silicate, so the electrostatic repulsive force is maintained in the diffusion layer. Therefore, the thickness of the electric double layer is thick. As a result, the primary particles become stable and weakly aggregate with each other. In contrast, at a higher content of ammonia, the ammonium ions shield all the negative charges on the surface of the aluminum silicate, so that van der Waals attraction dominates over electrostatic repulsive force. Therefore, the diffusion layer is greatly reduced, and the thickness of the electric double layer becomes thinner, as shown in Figure 5d. Thus, primary particles would be in an unstable state, and they would tend to aggregate with other neighboring particles [37,38,39,40,41]. This result is consistent with a previous report [42], in which as the concentration of electrolyte increased the aggregation rate increased because it shielded the electrostatic repulsion between the particles. Therefore, as the content of ammonia increased, the particles of aluminum silicate were more readily absorbed by one another, as the small particles were agglomerated and overall particle size increased.

**Figure 6:**
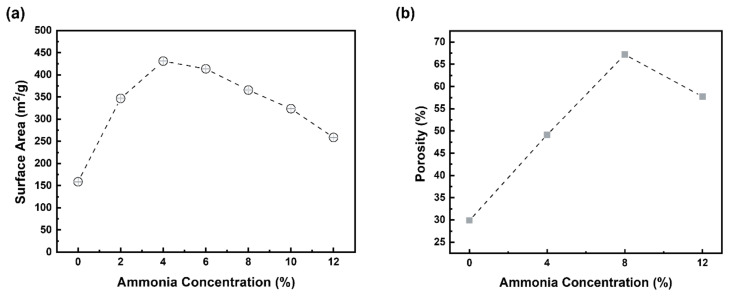
(**a**) BET-specific surface area, and (**b**) porosity of the synthesized aluminum silicate powder according to ammonia contents.

**Figure 7:**
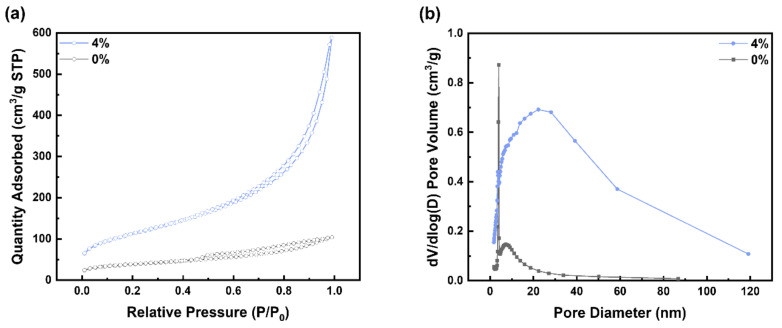
(**a**) N_2_ adsorption–desorption isotherms, and (**b**) BJH pore size distribution curve of AS-0 and AS-4.

Figure 6a graphically shows the change of specific surface area with variation in ammonia contents during the synthesis of aluminum silicate. It can be observed that the specific surface area of powder increased continuously with the increase in ammonia to 4%. Therefore, AS-4 had the largest specific surface area and exhibited a value more than twice that of AS-0, which had the lowest specific surface area. On the contrary, the specific surface area gradually decreased with the increasing ammonia contents in the solution. This can be explained by the variation of electrical double layer thickness with ammonia content, as mentioned earlier. Figure 6b shows the porosity according to the ammonia content of the synthesized powder. In the porosity, it can be seen that AS-4, AS-8, and AS-12 are larger than AS-0. Figure 7a shows the N_2_ adsorption–desorption isotherms which was performed to investigate the textural parameters of the porous powder. Both powders represent a type IV isotherm (according to IUPAC classification), which indicate that they have mesopores. Figure 7b shows the pore size distribution of AS-0 and AS-4. It was found that AS-4 has a larger pore size than AS-0.

**Figure 8:**
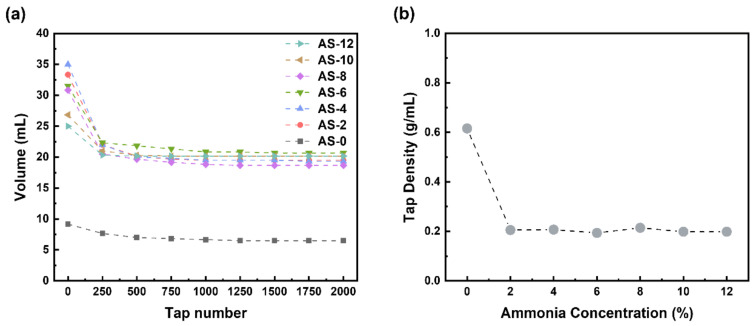
(**a**) tap volume and (**b**) tap density change of synthesized aluminum silicate powder according to ammonia contents.

To obtain the density of synthesized aluminum silicate powder, the tap density was measured, as shown in Figure 8. As can be seen in Figure 8a, the volume of AS-0 was a low value compared to the others and nearly constant with increasing the tap number, the powder of which had a very dense structure. However, the other powders had high volume and then the volume decreased as with increasing the tap number to 250 times, finally, they had a constant value up to 2000 times. The tap density for the powder with different ammonia content was calculated and represented in Figure 8b. The tap density of AS-0 was higher than the other powder. These results are attributed to the powder synthesized by two-step sol-gel method having a more porous structure compared to the powder synthesized by co-precipitation. It would be expected that the powder synthesized by the two-step sol-gel method has better filtration flowability due to low tap density than the powder synthesized by co-precipitation.

### 3.2. Adsorption Studies

To evaluate the adsorption performance for the synthesized aluminum silicate as the adsorbent, the adsorption capacity and removal efficiency of Pb (II) ion and methylene blue are measured. The adsorption capacity (*q_e_*) and removal efficiency (E) of Pb (II) ion and methylene blue in the synthesized aluminum silicate were calculated by the following equation [11,20,43].

(2) $$q_{e}=\frac{\left(c_{0\;}-c_{e}\right)V}{m}$$

(3) $$E=\frac{\left(c_{0}-c_{e}\right)}{c_{0}}\times 100\%$$

*c*_0_ and *c_e_* are initial concentration (mg/L), equilibrium concentration (mg/L), *m* represents the mass of aluminum silicate, and *V* represents the volume of Pb (II) ion and methylene blue solutions. The results are summarized in Table 4. As shown in Table 4, the adsorption capacity and removal efficiency were greatly increased with the addition of ammonia for the aluminum silicate.

There are various adsorption mechanisms between the adsorbent and the solution consisting of physisorption and chemisorption, such as electrostatic interaction, hydrogen bonding, ion exchange, coordination, and acid–base interaction [11,17]. Because a large number of silanol groups (Si-OH) and Si-O^−^ groups are formed on an aluminum silicate surface, the two groups are involved in the adsorption of Pb (II) ion and methylene blue [11]. The adsorption mechanism of the metal ion is that the Si-O^−^ group on the aluminum silicate surface has a strong electrostatic interaction with metal ions and is adsorbed. In the case of methylene blue, electrostatic interaction between the Si-O^−^ group on the surface of aluminum silicate and the cation of methylene blue and the hydrogen bond between the silanol group (Si-OH) and the amine group of methylene blue are present and adsorbed through it. Therefore, the electrostatic interaction and hydrogen bonding between aluminum silicate and metal ions, organic dyes contribute to adsorption and exhibit high adsorption capacity [11,17,24,43,44]. The maximum adsorption capacity of Pb (II) ions onto the AS-4 powder was 113.01 mg/g, which was over four times higher than that of the AS-0 powder (26.8 mg/g). In the case of methylene blue, the adsorption capacity of AS-4 powder was 149.5 mg/g, which was over seven times higher than that of the AS-0 powder (20.9 mg/g).

These results show that the presence of ammonia in two-step sol-gel method would be helpful to prepare the aluminum silicate powder having more higher specific surface area during the aqueous chemical process. Moreover, the AS-4 aluminum silicate powder, which had a higher specific surface area (430 m^2^/g) compared with conventional aluminum silicate, can be a potential adsorbent for removing heavy metal ions and organic dyes from aqueous solution.

## 4. Conclusions

The present work demonstrates the use of ammonia as a two-step sol-gel method in synthesizing aluminum silicate powder with a higher specific surface area. An aluminum silicate powder with amorphous was successfully synthesized with an aqueous chemical process regardless of different syntheses. However, by co-precipitation, the synthesized aluminum silicate powder had a relatively low specific surface area (158 m^2^/g) and the surface of the particle appeared to have a sharp edge, as though in a glassy state. On the other hand, in the case of forming an aluminum hydroxide sol by adding ammonia into aluminum precursor solution before dropping the sodium silicate, that is, when produced by a two-step sol-gel method, the synthesized aluminum silicate powder consisting of particles with a mesoporous structure had a large specific surface area of 430 m^2^/g. The aluminum silicate powders as adsorbents were characterized for their adsorption behavior towards Pb (II) ions and methylene blue in an aqueous solution performed in a batch adsorption experiment. The maximum adsorption capacity of Pb (II) ions onto the AS-4 powder was 113.0 mg/g, which was over four times higher than that of the AS-0 powder (26.8 mg/g). In the case of methylene blue, the adsorption capacity of AS-4 powder was 149.5 mg/g, which was over seven times higher than that of the AS-0 powder (20.9 mg/g). These results show that the presence of ammonia in two-step sol-gel method would be helpful to prepare the aluminum silicate powder having more higher specific surface area during the aqueous chemical process. Moreover, the AS-4 aluminum silicate powder, which had a higher specific surface area (430 m^2^/g), can be a potential adsorbent for removing heavy metal ions and organic dyes in fields such as electroplating, batteries, welding, and printed circuit boards where a lot of pollutants are discharged.

## Figures and Tables

**Table 1:** Notation of samples for preparing the aluminum silicate powder with different ammonia contents.

Sample Notation	Ammonia Content (Conc.)
AS-0	0%
AS-2	2%
AS-4	4%
AS-6	6%
AS-8	8%
AS-10	10%
AS-12	12%

**Table 2:** IR transmittance wavenumber (cm^−1^) and corresponding vibration bands of the synthesized aluminum silicate powder.

Wavenumber (cm^−1^)	Assignment	References
455	O-Si-O	[24]
580	Al-O_6_	[19,20]
715	Al-O-Si	[19,20]
1045	Si-O-Si	[19,20,25]
1400	Al-OH	[19,20]
1630	H-O-H	[11,19,20]
3440	O-H	[11,19,20]

**Table 3:** The particle size of the synthesized aluminum silicate powder according to ammonia contents.

Sample	Particle Size (μm)		
	d10	d90	Mean
AS-0	3.768	25.95	12.76
AS-2	5.505	22.59	13.15
AS-4	5.307	27.41	14.95
AS-6	5.197	28.10	15.12
AS-8	6.150	38.96	20.66
AS-10	6.157	39.19	20.14
AS-12	6.580	42.21	22.25

**Table 4:** Adsorption performance of the AS-0 and AS-4 powder for Pb (II) ion and methylene blue in aqueous solution.

	Pb (II) Ions		
	Concentration (ppm)	Adsorption Capacity (mg/g)	Removal Efficiency (%)
Initial	520.4		
0%	413.2	26.8	20.6
4%	68.4	113.0	86.9
	**Methylene Blue**		
	**Concentration (ppm)**	**Adsorption Capacity (mg/g)**	**Removal Efficiency (%)**
Initial	100		
0%	89.5	20.9	10.5
4%	25.2	149.5	74.8

## Data Availability

Not applicable.
